# HIV-Infected Mothers Who Decide to Breastfeed Their Infants Under Close Supervision in Belgium: About Two Cases

**DOI:** 10.3389/fped.2020.00248

**Published:** 2020-05-27

**Authors:** Nordin Bansaccal, Dimitri Van der Linden, Jean-Christophe Marot, Leïla Belkhir

**Affiliations:** ^1^Pediatric Infectious Diseases, Pediatric Department, Cliniques Universitaires Saint-Luc, Brussels, Belgium; ^2^Institut de Recherche Expérimentale et Clinique, UCLouvain, Brussels, Belgium; ^3^Internal Medicine Department, Cliniques Saint-Pierre, Ottignies-Louvain-la-Neuve, Belgium; ^4^Infectious Diseases, Internal Medicine Department, Cliniques Universitaires Saint-Luc, Brussels, Belgium

**Keywords:** HIV, breastfeeding, mother-to-child transmission, post-natal, breast milk, infection

## Abstract

**Introduction:** In most industrialized countries, human immunodeficiency virus (HIV) infection remains a formal contraindication to breastfeeding. However, for the past 9 years, the World Health Organization (WHO) has recommended, for developing countries, that mothers infected with HIV and treated by combined antiretroviral therapy (cART) should breastfeed their infants. HIV-infected women coming from developing countries and living in industrialized settings are increasingly expressing their natural desire to breastfeed. To avoid uncontrolled breastfeeding practices and reduce the risk of mother-to-child transmission of the virus, there is an urgent need to consider the wishes of these women.

**Discussion:** We report two cases in which specific guidelines were implemented in order to support the mothers' choice to breastfeed in Belgium. As a result of different prophylactic measures including antiretrovirals in mothers and infants and close follow-up, none of the infants were infected.

**Conclusions:** National or international recommendations for HIV-infected mothers who choose to breastfeed in industrialized countries remain unclear and discordant. There is an unmet need for experts to address this emerging issue and to develop an international consensus for the monitoring and prophylactic management of exposed-infants.

## Introduction

In 2017, The Joint United Nations Programme on HIV and AIDS estimated that 36.9 million people were living with the human immunodeficiency virus (HIV) (1), most of who were from low- and middle-income countries (LMIC). Mother-to-child transmission (MTCT) can occur during intra-uterine life, delivery or breastfeeding (2). The risk of transmission through breastfeeding is estimated at 0.064% per ingested liter, and at 0.028% per day of breastfeeding (3). Without intervention to prevent transmission, the risk of infection through breastfeeding varies between 13 and 48% (4–6).

In LMIC, the World Health Organization (WHO) recommends that HIV-infected mothers treated with combined antiretroviral therapy (cART) breastfeed their infants for 12–24 months. These recommendations are supported by an increased risk of morbidity and mortality due to infections (gastro-enteritis and pneumonia) and malnutrition in formula-fed babies. Access to drinking water is limited and formula milk is too expensive for these poorer populations (6–8).

In industrialized settings, MTCT of HIV in non-breastfed exposed infants is almost nil (9). For this reason, the international guidelines advise against breastfeeding in countries where mothers have access to clean water and affordable replacement feeding (infant formula). However, some groups of experts are currently revising their recommendations and, under certain optimal circumstances and close monitoring, are considering the option of breastfeeding for women who really wish to do so. Indeed, some HIV-infected women living in industrialized countries express a clear desire to breastfeed (7, 10–13) creating a new dilemma for healthcare professionals in those settings (14, 15).

We report the management of two pregnant HIV-positive women who expressed their desire to breastfeed. In addition to striving for normality, their motivations to breastfeed were mainly linked to cultural and social factors. The outcome for their two infants and the prevention of MTCT (pMTCT) strategy implemented are discussed below.

The first patient was a 36-years-old African woman living in Belgium. She was diagnosed with HIV and hepatitis B virus (HBV) infections at the age of 25. Her first child was born when she was 34 years old. He was breastfed in Congo and was not infected with HIV. A cART was initiated when she was 33 years. The adherence to treatment was difficult the patient found it very challenging to accept the fact that she was infected with HIV. It was therefore tough for her to take several medications for disease she did not acknowledge. Consequently, the medical team including doctors, nurses and psychologists, followed the patient regularly and her initial regimen was simplified to a single molecule Emtricitabine/Tenofovir/Elvitegravir/Cobicistat (FTC/TDF/EVG/COBI). She eventually accepted the illness and followed her treatment correctly. The viral load (VL) was undetectable after 1 year of treatment. The patient became pregnant the following year. The plasma viremia was suppressed throughout pregnancy and at delivery. The patient gave birth to a term baby girl by vaginal delivery. Our medical team strongly advised against breastfeeding several times. Nevertheless, the patient decided to exclusively breastfeed her newborn baby for 4 months. The reasons she evoked included social pressure, work constraint, and the fear of being rejected by her community. To support breastfeeding, the mother was closely followed by a multidisciplinary team including infectiologists, pediatricians, nurses, midwives, psychologists, and social workers. The team's role was to support, inform and answer all of the mother's questions in order to ensure the safety of her breastfeeding for her child. Our approach was inspired by an excellent English brochure provided by Saint-Mary's Hospital Family Clinic. Close clinical and biological follow-up was, therefore, organized. The infant was given a dual cART with Lamivudine (3TC) and Zidovudine (AZT) from birth until 1 month after weaning. The mother remained on cART with undetectable VLs during this period. Blood and milk VLs remained undetectable during the breastfeeding period. Monthly HIV-1 DNA and RNA PCR in the infants's blood remained negative up to 1 month after weaning. The child was considered as non-infected when HIV antibody testing was negative at 18 months.

The second patient was a 35-years-old African woman living with HIV. She had been treated for 5 years when she became pregnant with her second child. The VL remained undetectable throughout pregnancy. The patient gave birth to a baby girl by cesarean section due to prolonged labor. The mother had not been able to breastfeed her first child and wished to breastfeed her second child arguing that she knew of HIV-positive women living in Congo who had breastfed. Although the medical team advised the patient against breastfeeding and encouraged feeding exclusively with formula milk, she began breastfeeding. However, formula milk supplements had to be temporarily introduced when the newborn lost significant weight and was at risk of dehydration and hypernatremia. From birth onwards and during the mixed feeding period, the baby was given a triple cART with AZT, 3TC, and Nevirapine (NVP) for a few days only and subsequently bitherapy (3TC-AZT) when he reached an adequate weight and breastfeeding was again exclusive. The mother stopped breastfeeding when the infant was 5 months old. HIV DNA nucleic acid tests were performed every month during this period and for a month after weaning and were all negative. The milk and mother's blood VLs remained undetectable during the breastfeeding period. The infant was also non-infected with negative HIV antibody testing at 18 months.

## Discussion

In most LMIC, breastfeeding is the standard mode of nutrition for infants and formula-feeding remains relatively unusual. An English study describes the difficulty in applying national or international recommendations contra-indicating breastfeeding to certain women who have migrated from countries where breastfeeding is the norm. For those women, the abstention of breastfeeding can be difficult emotionally and psychologically (16). The current migratory flows from the South to the North are leading to increasing confrontations between medical recommendations and mothers' wishes to breastfeed.

Breastfeeding is an indisputable human right and its benefits on immunity, nutrition and attachment between the mother and infant are well-recognized. Breastfeeding also plays an important role in the mother's health. For example, it contributes to uterine involution, weight loss, reduction in the risk of postpartum depression, lactational amenorrhea or reduction in the risk of ovarian cancer and endometrium. Breastfeeding also has the advantage of being free and available immediately. However, in the case of the mother's HIV infection in Western countries, artificial milk is often fully reimbursed or donated by hospitals or milk companies. Given all the benefits for the child and the mother, the contraindication of breastfeeding for women infected with HIV can be experienced as a paradoxical message (17, 18). In Belgium, many public health campaigns promote breastfeeding. This commitment of society can sometimes indirectly stigmatize these mothers who cannot breastfeed for “medical reasons.” This stigma can also extend to family and relatives. Indeed, breastfeeding varies according to beliefs traditions and religions. In some communities, breastfeeding is a social norm.

Several research groups have investigated methods that would allow HIV-infected mothers to safely breastfeed their infants. However, in addition to the risk of HIV transmission during breastfeeding, infants are exposed to repeated blood tests and to potentially harmful prophylactic antiretroviral therapy. Studies have also shown that cART pass into breast milk (19) but their adverse effects, namely on psychomotor and cognitive development, have not been clearly established (20, 21).

According to a meta-analysis in LMIC, MTCT varies between 0.5 and 7.9% when the mother breastfeeds for up to 6 months. The overall risk was evaluated at 3.5%. This heterogeneity can be explained by the different methodologies used in the studies. Despite some statistical inconsistencies between the studies, the overall risk was directly correlated with the duration of breastfeeding (22). Transmission rates are certainly not transposable to industrialized countries and no studies exist in these settings.

One of the major risk factors for HIV transmission during breastfeeding is the presence of virus in both plasma and maternal milk (23). These two factors are directly correlated (24, 25). In developing countries, cART reduces the risk of post-natal transmission to <2% (26, 27). In order to prevent the risk of transmission into breast milk, it is usually recommended that the plasma VL should be kept below the threshold of 100 copies/ml (28). Reducing the viral load of breast milk by administering cART to breastfeeding mothers may further reduce post-natal transmission of HIV to infants who are exclusively breastfed (29). However, the risk of HIV transmission is not totally eliminated (7) and may occur despite a very low level of HIV RNA copies in blood or breast milk (24, 26). Vertical MTCT has been reported even in cases where the viral load was <100 copies/mL in maternal plasma (30). Indeed, there persists a theoretical risk of transmission from infected epithelial cells present in the breast milk of mothers on cART (31). Therefore, there is no precise viral threshold under-which the lack of transmission is guaranteed (23).

Adherence to cART is crucial in reducing VL in plasma and subsequently in breast milk (23, 28, 32). Non-adherence to treatment may be related to many factors including lack of access to treatment, stigma, discrimination, forgetfulness and drug treatment changes. Social norms, socio-economic status, and lack of knowledge about the disease and treatment are the main barriers encountered when treating HIV patients in developing countries (33). Education about transmission, HIV prevention, and efficacy of cART are needed in order to increase adherence to treatment and follow-up (34). This is especially important in the postpartum period as studies have shown a decrease in adherence to treatment after delivery (35). Psychological counseling and support for family members, spouses, and the community promote greater participation and adherence to treatment (36).

To date, due to a low theoretical risk of transmission, most recommendations in industrialized settings do not recommend breastfeeding for women with HIV. However, a new approach is emerging. In their October 2018 updated guidelines, the European AIDS Clinical Society discourages breastfeeding in case of HIV infection, but recommends close monitoring of children born to women who wish to breastfeed despite this advice. The British HIV Association 2018 guidelines also advise against breastfeeding but recommend support for women who wish to breastfeed only if VL is undetectable. Maternal VL should ideally be undetectable throughout the pregnancy, and certainly 3 months before delivery. Infants must then undergo clinical and biological monitoring within 48 h of birth, at the age of 2 weeks, every month during the breastfeeding period and for up to 2 months after weaning. HIV antibody testing should be done between 18 and 24 months. Ideally, the breastfeeding period should be as short as possible. Breastfeeding must be stopped in the case of mastitis, or if the infant shows symptoms of gastroenteritis. Women who do not meet these safety criteria should be informed of the risks and be advised to discontinue breastfeeding (12).

For our second patient, we chose the option of triple therapy and mixed breastfeeding because of an 11.5% loss of body weight when the newborn was 2 days old. Indeed, mixed breastfeeding has previously been associated with an increased risk of MTCT, irrespective of the plasma or breast milk VL. This association was demonstrated in the absence of optimal cART (5, 37, 38). This is probably due to a decrease in the immunological capacity of the milk and the ability of the child to resist the virus (38–40). Also, intestinal permeability decreases more rapidly in infants fed with artificial milk than human milk (41). Note that other elements can disrupt intestinal permeability and facilitate the passage of the virus, such as gastroenteritis or antibiotics, which modify the intestinal flora. However, in 2016, Chikhungu et al. demonstrated, in a large review of eighteen studies between 2005 and 2015, that there is no significative difference between exclusive breastfeeding and mixed feeding in the risk of transmission in infants of women infected with HIV on cART (42).

In industrialized countries, there is no studies that shows an increased risk of transmission in the case of mixed breastfeeding compared to exclusive breastfeeding when VL is undetectable. Our team have had a protectionist attitude toward children given the lack of data in Western countries, however the assumed risk being negligible or even zero, this attitude can be discussed.

US recommendations suggest treating breastfed children with AZT for 6 weeks and/or 3 doses of NVP at 48 h of birth, 48 h after first dose, and 96 h after second dose (11). Some authors, recommend pursuing prophylaxis up to 1 month after weaning (11) as we chose to do in the two cases presented. This choice was justified by the increased risk of transmission when breastfeeding is interrupted (43, 44).

The Federal Commission for Sexual Health (Swiss interim recommendations) do not advocate infant prophylactic treatment when the viral load is <50 copies/ml (45) in order to avoid treatment-related adverse events, such as mitochondrial toxicity (3TC, AZT) and hematologic toxicity (AZT). However, this approach could be discussed in view of the theoretical residual risk of transmission by infected epithelial cells, the uncertainty of maternal adherence to cART and issues regarding factors that may promote intestinal passage of the virus, such as gastroenteritis or mixed feeding.

## Conclusions

Breastfeeding by HIV-infected mothers remains a complicated dilemma for health professionals. Exclusive artificial breastfeeding remains the most reliable and safe way to prevent transmission in developed countries. Few studies discuss the consequences of breastfeeding on children when mothers are infected with HIV in industrialized countries. The current recommendations advise on supporting the decision of HIV-infected mothers who really want to breastfeed but remain vague about the strategies that should be implemented. There is a clear unmet need to clarify these issues in order to best accompany HIV-infected women in their choice to breastfeed while ensuring the safety of their infants.

## Ethics Statement

Written informed consent was obtained from the individual(s) for the publication of any potentially identifiable images or data included in this article.

## Author Contributions

NB has written the first draft. DV and LB substantially work on the initial draft and further versions along with NB. J-CM has participated by giving his input and comments on the manuscript.

## Conflict of Interest

The authors declare that the research was conducted in the absence of any commercial or financial relationships that could be construed as a potential conflict of interest.
